# Serum Amyloid Beta Precursor Protein, Neurofilament Light, and Visinin-like Protein-1 in Rugby Players: An Exploratory Study

**DOI:** 10.3390/sports10120194

**Published:** 2022-11-29

**Authors:** Jessica E. Morgan, Sarean A. Gaynor-Metzinger, Steven D. Beck, Iustin C. Scobercea, India J. Austin, Hannah E. Blankenship, Julien S. Baker, Allan Knox, Jorge M. Serrador, Matthew J. Rogatzki

**Affiliations:** 1 Department of Public Health and Exercise Science, Appalachian State University, Boone, NC 28608, USA; 2 Department of Kinesiology, University of Wisconsin-Madison, Madison, WI 53703, USA; 3 Cardio-Renal Physiology Laboratory, Department of Biology, Appalachian State University, North Carolina Research Campus, Kannapolis, NC 28081, USA; 4 College of Osteopathic Medicine, Liberty University, Lynchburg, VA 24515, USA; 5 Centre for Health and Exercise Science Research, Hong Kong Baptist University, Kowloon Tong, Hong Kong, China; 6 Exercise Science Department, California Lutheran University, Thousand Oaks, CA 91360, USA; 7 The MARCS Institute for Brain, Behaviour and Development, Western Sydney University, Westmead, NSW 2751, Australia; 8 Rehabilitation and Movement Sciences, School of Health Professions, Department of Pharmacology, Physiology and Neuroscience, New Jersey Medical School, Rutgers, The State University of New Jersey, Newark, NJ 07102, USA

**Keywords:** concussion, biomarkers, amyloid beta precursor protein, visinin-like protein-1, neurofilament light, rugby

## Abstract

Concussion diagnosis is difficult and may be improved with the addition of a blood-based biomarker that indicates concussion. The purpose of this research was to investigate the capability of serum amyloid beta precursor protein (APP), neurofilament light (NfL), and visinin-like protein-1 (VILIP-1) to distinguish athletes who were diagnosed with a concussion pitch-side. An observational cross-sectional study design was used to replicate sideline concussion diagnosis. Subjects included mutually exclusive pre-match (*n* = 9), post-match (*n* = 15), and SRC (*n* = 7) groups. Six paired pre-and post-match subjects were analyzed for APP. APP increased significantly from pre-match (mean = 57.98 pg·mL^−1^, SD = 63.21 pg·mL^−1^) to post-match (mean = 111.37 pg·mL^−1^, SD = 106.89 pg·mL^−1^, *p* = 0.048) in the paired subjects. NfL was lower in the SRC group (median = 8.71 pg·mL^−1^, IQR = 6.09 pg·mL^−1^) compared to the post-match group (median = 29.60 pg·mL^−1^, IQR = 57.45 pg·mL^−1^, *p* < 0.001). VILIP-1 was higher in the post-match group (median = 212.18 pg·mL^−1^, IQR = 345.00 pg·mL^−1^) compared to both the pre-match (median = 32.63 pg·mL^−1^, IQR = 52.24 pg·mL^−1^), *p* = 0.001) and SRC (median = 30.21 pg·mL^−1^, IQR = 47.20 pg·mL^−1^), *p* = 0.003) groups. APP, NfL, and VILIP-1 were all able to distinguish between pre-match and post-match groups (AUROC > 0.700) but not from the SRC group (AUROC < 0.660). Our results show that APP, NfL, and VILIP-1 were not helpful in differentiating concussed from non-concussed athletes pitch-side in this study.

## 1. Introduction

Sports-related concussion (SRC) is defined as a “traumatic brain injury induced by biomechanical forces” [1]. Repeated concussions are shown to be associated with the development of chronic traumatic encephalopathy (CTE) [2], and include symptoms of depression, headache, memory loss, concentration difficulties, explosive behavior, and gait disturbance [3]. Concussion incidence is of great concern in rugby players. For example, in the 2020–2021 season, the rugby football union reported a concussion incidence of 22.2 concussions per 1000 h, which was the highest incidence on record [4]. Additionally, a recent study indicated that former international rugby union players are at greater risk of developing neurodegenerative diseases compared to members of the general population [5]. Accurate diagnosis of SRC is important in allowing athletes to return to play if they did not have concussion but also to remove athletes from play if a concussion did occur to allow proper brain healing. Failure to remove athletes from play following a concussion can have catastrophic consequences such as second impact syndrome. Second impact syndrome occurs as a result of experiencing a subsequent concussive trauma following an initial concussion and can result in permanent brain damage or even death [6]. Diagnosis of SRC is difficult due to dependence on reported symptoms, overlapping symptoms caused by other common conditions, and an absence of validated, objective tests [7]. Agreeing with this difficulty, a survey of certified athletic trainers (ATCs) found their self-efficacy in SRC evaluation to be moderate [8]. This moderate self-efficacy is not due to lack of professional skill on the part of ATCs, but poor confidence in the current SRC diagnostic tools available. High self-efficacy is pertinent in ensuring the safety of concussed individuals [8]. Adding a blood-based biomarker to the armamentarium of tools for SRC diagnosis will enhance the precision of diagnosing SRC reducing the contribution of self-efficacy to SRC diagnosis among ATCs.

Therefore, we conducted this study to investigate three proteins that have potential as SRC diagnostic biomarkers. We decided to investigate amyloid beta precursor protein (APP) in our study as it has never been investigated in the blood of concussed athletes. However, Cairelli et al. determined that APP may be a useful biomarker of brain injury [9], while Boutté et al. also suggested that APP may be worth investigating in athletes experiencing concussion and subconcussion [10]. Visinin-like protein-1 (VILIP-1) was also chosen since little research has been conducted on this protein in concussed athletes, and the research that has been conducted showed that VILIP-1 peaked one-hour post injury [11], indicating it may be useful for sideline diagnosis. Neurofilament light (NfL) was included in this study as it does not appear to be affected by exercise alone [12] and has shown promise as a potential biomarker for diagnosing concussion [13].

Increased levels of both APP and NfL indicate axonal injury, and diffuse axonal injury may occur as a result of concussion [14]. Therefore, we hypothesized that both serum APP and NfL would increase significantly post-SRC in comparison to the pre-match and post-match groups. VILIP-1 was found to increase in concussed athletes so we hypothesized that serum VILIP-1 would be elevated significantly following SRC in contrast to the pre-match and post-match groups.

## 2. Materials and Methods

### 2.1. Study Population and Design

The Rutgers University (New Brunswick, NJ, USA) and Appalachian State University (Boone, NC, USA) Institutional Review Board (IRB) approved this study. Written informed consent was obtained from athletes in a research tent before participation in the study. The research study was an observational cross-sectional design.

Recruitment of rugby players occurred at individual collegiate club rugby matches during the Appalachian State University Men’s club rugby team’s Fall 2019 season and at the 2019 annual Can-Am rugby tournament in Upstate New York. Rugby players attending the Can-Am tournament ranged from amateur to collegiate level. Rugby athletes were divided into pre-match, post-match, and SRC cohorts. This study was designed to collect blood from the pre-match group soon before their match, from the post-match group immediately after finishing their match, and from the SRC group immediately after concussion diagnosis by a certified athletic trainer (ATC).

### 2.2. SRC Diagnosis and Grouping of Rugby Players

**Figure 1:**
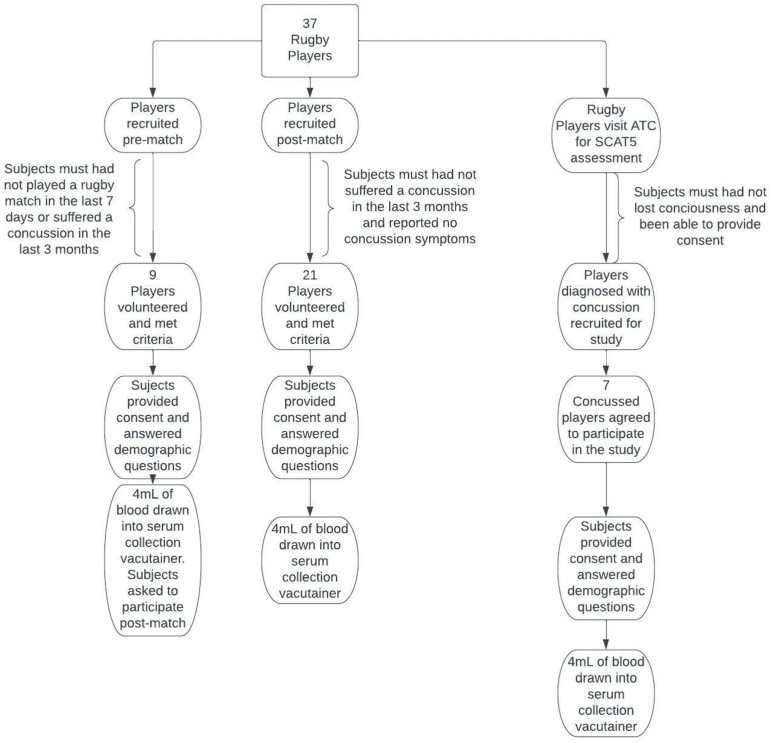
Schematic of recruitment methods. SRC = Sports-related concussion; SCAT5 = sport concussion assessment tool-5th edition.

In total, 37 rugby players volunteered for this study. Of the 37 participants, nine donated blood pre-match, 21 donated blood post-match, and 7 donated blood following SRC. Pre-match subjects were enrolled in the study through inquisition of players before match play if they would like to join the research study. Players could not participate as pre-match subjects unless they had not played rugby in the past seven days and had not had a concussion in the previous three months. Players participating pre-match were also asked to participate in the post-match group to allow for paired samples. The post-match group of rugby players were recruited by asking players not reporting any signs or symptoms of concussion to join the research immediately after their match, and had not experienced a concussion in the previous three months. Players were included in the SRC group if on-site athletic trainers diagnosed them with an SRC using the Sport Concussion Assessment Tool—5th edition (SCAT5) [15]. Subjects were excluded from the SRC group if they had lost consciousness or were unable to provide consent. While ATCs overall report moderate self-efficacy for SRC diagnosis, the ATCs involved with this study each had approximately 10 years of professional experience. Since evaluation by an ATC is currently accepted as the best way of diagnosing concussion pitch side, ATC assessment was used as the standard diagnostic tool for this study. A flow chart illustrating player recruitment procedures is depicted in Figure 1.

### 2.3. Collection and Processing of Blood

Each subject donated a four milliliter venous blood sample obtained from a prominent vein in the antecubital space. The venous blood draws took place within a tent located pitch-side of the rugby match. Blood was collected in a silicone-coated vacutainer and held at ambient temperature for at least 30 min but no more than 60 min allowing the blood to clot. Following the clotting process, the blood was centrifuged using a StatSpin Express 3 portable centrifuge (Beckman Coulter, Inc., Brea, CA, USA) for 5 min at 5600 revolutions per minute (2685 relative centrifugal force). This caused separation of the serum from the red blood cells. After centrifugation, the serum was removed and stored in a −80 °C portable freezer (model ULT25NEU, STIRLING ULTRACOLD A division of Global Cooling, Inc., Athens, OH, USA) until transfer to a permanent −80 °C freezer until biomarker analysis.

### 2.4. Biomarker Analysis

APP serum concentration ([APP]) was determined using a human APP ELISA-96-well plate assay kit (OKEH02647, Aviva Systems Biology Corporation, San Diego, CA, USA). The detection range of the assay was 15.6–1000 pg·mL^−1^, with a limit of detection (LOD) less than 10 pg·mL^−1^. A linear regression analysis was used to create a standard curve (R^2^ = 0.9963). Average intra-assay coefficient of variation was 18.31%.

NfL serum concentration ([NfL]) was determined using a human NfL ELISA-96-well plate assay kit (MBS765857, MyBioSource, Inc., San Diego, CA, USA). The detection range of the assay was 15.625–1000 pg·mL^−1^, with a limit of detection (LOD) less than 9.375 pg·mL^−1^. A 4-parametric logistic regression analysis was used to create a standard curve (R^2^ = 0.9993). Average intra-assay coefficient of variation was 27.12%.

VILIP-1 serum concentration ([VILIP-1]) was determined using a human VILIP-1 ELISA-96-well plate assay kit (MBS760977, MyBioSource, Inc., San Diego, CA, USA). The detection range of the assay was 31.2–2000 pg·mL^−1^, with a limit of detection (LOD) less than 18.75 pg·mL^−1^. A 4-parametric logistic regression analysis was used to create a standard curve (R^2^ = 0.9935). The average intra-assay coefficient of variation was 18.15%.

Standards and samples were analyzed in duplicate. All samples for each assay were run on one plate. Absorbances for the assays were read at 450 nm using an Eon spectrophotometer (BioTek Instruments, Inc., Winooski, VT, USA).

### 2.5. Statistical Analysis

A G*Power (Heinrich Heine University Düsseldorf, Düsseldorf, Germany) analysis using a 0.8 power and 0.7 effect size was conducted using data obtained from previous research on NfL [12] and VILIP-1 [11] since similar research has been conducted in the past on these two proteins, whereas no previous research looking at APP in concussed and non-concussed athletes has been conducted. The G*Power analysis showed the need for 8 subjects per group. Although we only reached 7 subjects in the SRC group, we continued with statistical analysis due to the challenges of obtaining consent and venous blood draw soon after concussion as well as stopping of rugby play due to the COVID-19 pandemic. A large effect size was used to highlight the need for credible biomarkers indicating concussion. If the effect size is small for a biomarker, it is less likely the biomarker would be helpful in improving SRC diagnosis. However, it is possible that several biomarkers with a small effect size may be combined to accurately diagnose concussion, which would require a larger subject pool than included in this study.

To compare median age, body mass, years of play, concussion history, serum APP, serum NfL, and serum VILIP-1 among the three groups of rugby players a non-parametric Kruskal–Wallis test was used since the sample sizes in each group were unequal and since there was a lack of normality as assessed by a Shapiro–Wilk normality test. When significant differences were found among protocols a Dunn–Bonferroni post hoc analysis was used to determine between which groups those differences occurred. Normal distribution of the paired pre- and post-match APP data was determined using a Shapiro–Wilk normality test. Paired data for NfL and VILIP-1 were not analyzed due to low sample size (*n* = 2). Following confirmation of normality, A paired T-test was used in comparing pre-match to post-match serum APP levels. To determine if APP, NfL, or VILIP-1 could distinguish between groups an Area under the receiver operating characteristic curve (AUROC) was used. A 95% confidence interval (CI) was used to assign bounds of expected discrepancy between sample and population means. Partial eta squared (η^2^) was used to calculate effect size among groups, Cohen’s D (d) was used to calculate effect size between the paired pre- and post-match APP data, and a 95% confidence interval (CI) was used to assign bounds of expected discrepancy between sample and population means. Alpha was set at 0.05 in order to determine statistical significance.

## 3. Results

Subjects included adult (age 18–63 years) Caucasian male (*n* = 35) and female (*n* = 2) rugby players participating in rugby tournaments and matches during the summer and fall of 2019. No differences were found in age (*p* = 0.119, η^2^ = 0.098), body mass (*p* = 0.345, η^2^ = 0.043), years of play (*p* = 0.143, η^2^ = 0.376), or concussion history (*p* = 0.082, η^2^ = 0.217) among groups despite the post-match group having higher age and more years of play than both the SRC and pre-match groups, while the SRC group had a greater history of concussions than both the pre-match and post-match groups. Since we had two females in our data set, we analyzed the data both with and without the inclusion of female data. Because removing the females from analysis did not change the statistical results, we included their data in all analyses. Four subjects over the age of 50 (only one of those subjects were above age 60, at 63 years old) were in the post-match group causing the large standard deviation in age. Since age, especially over the age of 60, has shown to increase NfL levels [16], a separate statistical analysis was conducted after the removal of these four subjects. However, this did not change the statistical outcomes for any of the data. Therefore, we included these four subjects in our analyses. Demographic information for the pre-match, post-match, and SRC groups can be found in Table 1. The pre-match group was comprised of players who provided blood immediately prior to a match, the post-match group was comprised of players who provided blood an average of 43.35 (SD 60.02) minutes after finishing a match, and the SRC group was comprised of players who provided blood an average of 50.43 (SD 60.42) minutes after sustaining an SRC. Descriptive statistics for APP (Tables S1 and S2), NfL (Table S3), and VILIP-1 (Table S4) can be found in the Supplementary Materials.

### 3.1. Differences in Subject Numbers

During the assay process, it was discovered that there was enough serum from some subjects to perform only one assay. Therefore, for APP there were 21 subjects in the post-match group whereas there were 15 subjects in the NfL and VILIP-1 post-match groups. The number of subjects in the pre-match group (*n* = 9) and SRC group (*n* = 7) were the same for all biomarkers. Due to the difference in numbers of post-match data, we had paired data for six subjects in APP analyses but only paired data for two subjects in the NfL and VILIP-1 analyses. None of the concussed subjects had donated pre-match blood samples.

### 3.2. APP

**Figure 2:**
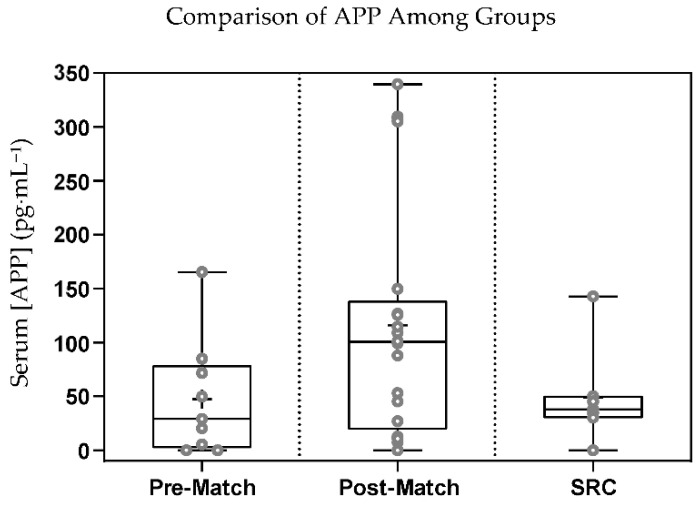
Box and whisker plot showing the serum concentration of amyloid beta precursor protein (APP) among the three groups of subjects. The ‘+’ represents mean serum APP concentration and the line through the box represents median serum APP concentration. Interquartile ranges were calculated using inclusive median, *p* = 0.134, degrees of freedom = 2, η^2^ = 0.132.

**Figure 3:**
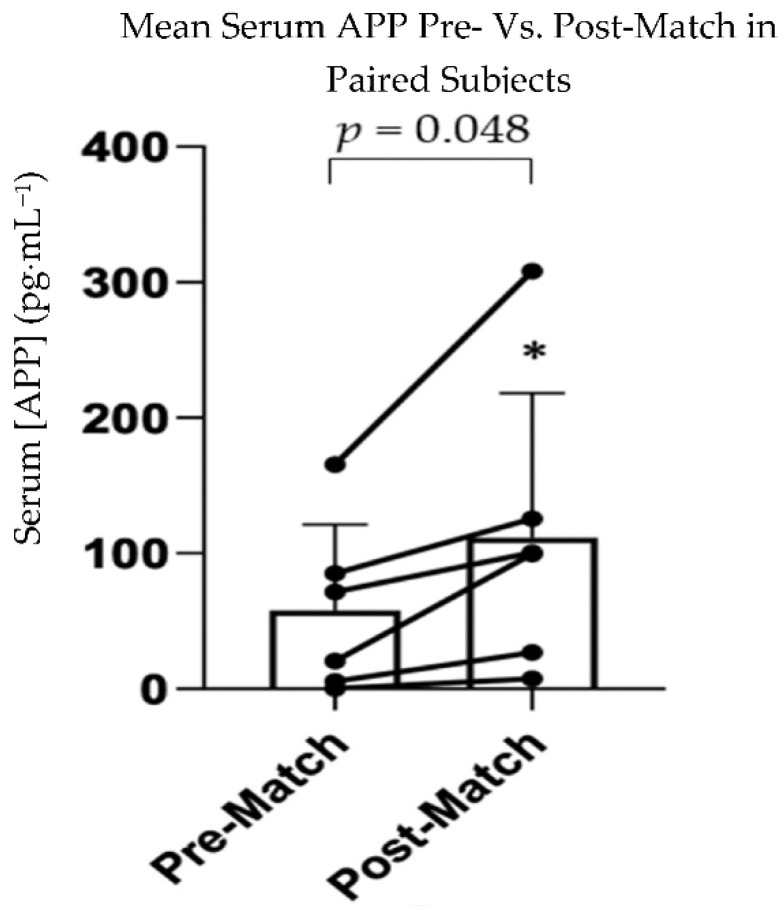
Bar graph showing mean paired data for paired pre-match and post-match amyloid beta precursor protein (APP) serum concentration. Connected dots show data for each individual subject (*n* = 6). * = statistically significant compared to pre-match, *p* = 0.048, d = 1.07, degree of freedom = 5.

The Kruskal–Wallis test did not show any significant difference among the three groups for APP (*p* = 0.134, η^2^ = 0.132) (Figure 2). Paired T-tests showed a significantly greater concentration of serum APP in the post-match compared to the pre-match group (*p* = 0.048, d = 1.07) (Figure 3). AUROC values, 95% confidence intervals, and *p*-values for all group comparisons can be found in Table 2. The AUROC comparing SRC to the post-match (AUROC = 0.330) and pre-match (AUROC = 0.556) groups showed that APP did not distinguish between athletes sustaining an SRC and those playing a contact sport or athletes prior to match play in this study. The AUROC comparing the post-match to pre-match group (AUROC = 0.709) was not statistically significant.

### 3.3. NfL

**Figure 4:**
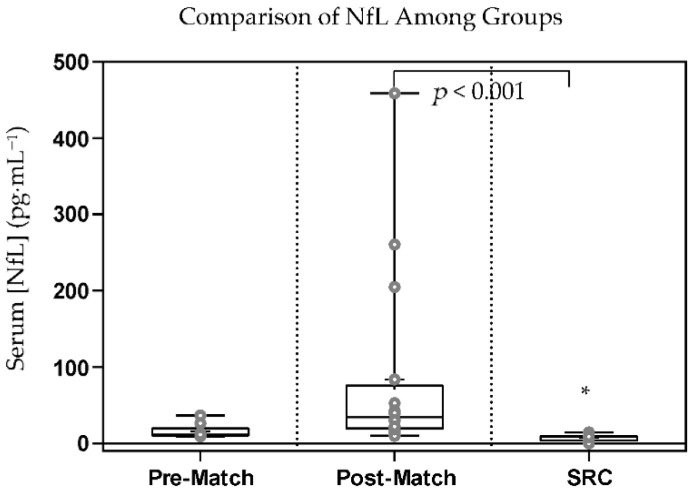
Box and whisker plot showing the serum concentration of neurofilament light (NfL) among the three groups of subjects. The ‘+’ represents mean serum NfL concentration and the line through the box represents median serum NfL concentration. Interquartile ranges were calculated using inclusive median. * = statistically significant compared to post-match, *p* < 0.001, degrees of freedom = 2, η^2^ = 0.150.

Significant differences among groups were found for NfL (*p* < 0.001, η^2^ = 0.150) using the Kruskal–Wallis test. This significant difference was found comparing SRC to post-match (*p* < 0.001) (Figure 4). No significant differences were found for NfL when comparing pre-match to SRC (*p* = 0.271) or post-match (*p* = 0.052). The AUROC comparing SRC to the post-match (AUROC = 0.029), and pre-match (AUROC = 0.127) groups showed that NfL did not distinguish between athletes sustaining an SRC and athletes pre- or post-match. However, the AUROC comparing the post-match to pre-match showed NfL (AUC = 0.852) as a good test for distinguishing between athletes after playing a contact sport and athletes prior to playing a contact sport (Table 3).

### 3.4. VILIP-1

**Figure 5:**
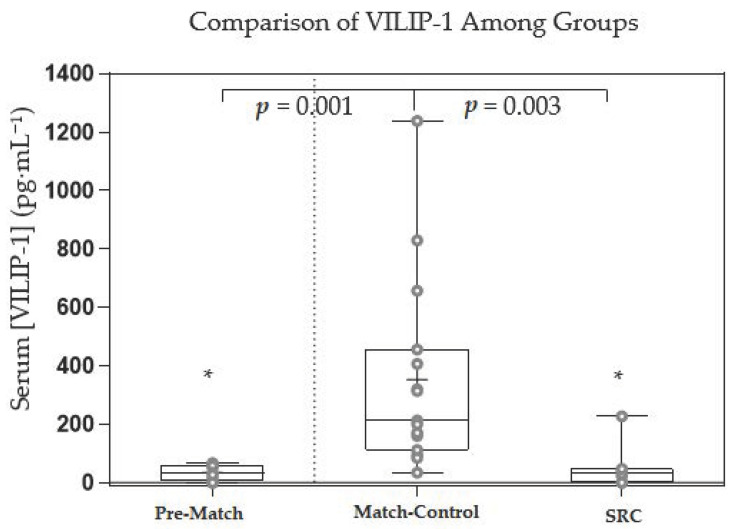
Box and whisker plot showing the serum concentration of visinin-like protein-1 (VILIP-1) among the three groups of subjects. The ‘+’ represents mean serum VILIP-1 concentration and the line through the box represents median serum VILIP-1 concentration. Interquartile ranges were calculated using inclusive median. * = statistically significant compared to post-match, *p* < 0.005, degrees of freedom = 2, η^2^ = 0.321.

Significant differences among groups for VILIP-1 (*p* < 0.001, η^2^ = 0.321) were found using the Kruskal–Wallis test. The significant difference was found when comparing post-match to SRC (*p* = 0.003) and pre-match (*p* = 0.001) (Figure 5). No significant difference was found when comparing SRC to pre-match (*p* = 1.00). The AUROC comparing SRC to the post-match (AUROC = 0.095) and pre-match (AUROC = 0.444) groups show that VILIP-1 did not distinguish between athletes sustaining an SRC and athletes pre- or post-match (Table 4). The AUROC comparing post-match to pre-match showed VILIP-1 (AUROC = 0.970) as an excellent test for distinguishing between athletes after playing a contact sport and athletes prior to playing a contact sport.

Combining APP, NfL, and VILIP-1 did not improve the AUROC for any of the group comparisons.

## 4. Discussion

The purpose of this research was to investigate if serum APP, NfL, and VILIP-1 in blood collected pitch-side could distinguish rugby players who suffered anSRC from pre-match and post-match control athletes. All data were collected during summer and fall of 2019. Further data collection was halted in the spring of 2020 due to the COVID-19 pandemic and shuttering of all rugby play. During this time, research found that COVID-19 infection may cause increased levels of brain specific biomarkers [18,19,20]. Therefore, we decided to analyze this COVID-19 naïve cohort separate from any post pandemic cohorts to avoid the confounding variable of COVID-19 infection.

Following a literature search, it appears that this is the first study to investigate APP in human blood, specifically to assess SRC. Our results show that APP was not different among the three groups; although the AUROC indicated that APP may be able to distinguish between pre- and post-match athletes. Based on previous research in CSF, we speculate that the immediate blood draw in the post-match and SRC groups may not have allowed sufficient time for an increase in APP. For example, significant increases in APP were not found in CSF until at least 2 days after severe traumatic brain injury [21]. This timing requirement is also suggested in our study by serum APP being greater (although not significantly greater) in post-match individuals, who likely had longer playing time during each match (thus longer time for APP to accumulate in blood rather than greater axonal injury), compared to those with SRC who were removed from the match early.

Interestingly, our results did show that APP was significantly greater post-match compared to pre-match when analyzing paired data. Serum APP levels were greater post-match in every athlete and an average of 1.9-fold higher post-match compared to pre-match. This finding is concerning as increased levels of APP have been suggested to result in a greater likelihood of β-amyloid deposits in the brain [22]. Beta-amyloid deposits are associated with Alzheimer’s disease (AD) [23] and CTE [24], both of which have been linked to brain injury from sport. Therefore, increases in APP levels caused by participation in a contact sport may place contact sport athletes at an elevated risk of developing β-amyloid deposits in the brain and future development of AD or CTE. However, the increase in APP may also be the result of a disrupted blood–brain barrier due to rugby play [25] and/or exercise affecting glymphatic flow [26]. In addition, due to the low subject numbers in our study, and lack of comparison to non-contact athletes, we are not suggesting that these data indicate rugby players are at a higher risk of developing these neurological diseases; rather, we believe these data suggest further investigation of this protein in contact athletes. Additional research including non-contact sport athletes and monitoring the magnitude and number of impacts in contact sport athletes may help elucidate if subconcussive injury [27] is causing the increase in serum APP levels.

To our knowledge, three studies have investigated serum or plasma NfL soon after SRC (1 h post-SRC) to determine its effectiveness in side-line concussion diagnosis [12,28,29]. All three of these studies have found similar results as our study when comparing NfL in athletes experiencing SRC 1 h post injury compared to contact controls [28,29]. Shahim et al., 2017, found that NfL could distinguish between hockey athletes with rapidly resolving postconcussion symptoms (PCS) and those experiencing prolonged PCS [28]. However, serum NfL was not significantly higher than non-athletic controls at 1 h post SRC, but was significantly higher at 12 h post SRC [28]. Shahim et al., 2018, again found that NfL may be useful in identifying individuals with prolonged PCS in hockey players, and also found that NfL one-hour post-concussion was significantly higher than baseline, gymnastic controls, and healthy controls [12]. However, no statistical comparison was made between the concussed and friendly hockey game control group [12]. Laverse et al., 2020, found significant increases in NfL 1 h post-mTBI and at the 3–10 day time point compared to baseline. However, when comparing mTBI subjects to contact controls there was no difference in NfL at any time point [29]. Our results, along with these other studies, suggest that NfL may not be a good biomarker to distinguish concussed athletes from contact controls pitch-side, but may be a good indicator of prolonged PCS risk. The National Collegiate Athletic Association (NCAA) and the US Department of Defense Concussion Assessment, Research, and Education (CARE) Consortium investigated serum NfL in concussed contact sport athletes compared to contact controls [30] as well as military cadets experiencing concussion compared to contact control cadets [31]. Results from these studies reported no significant difference in serum NfL at the acute time point (<6 h), 24–48 h post-injury or at the asymptomatic or return-to-play timepoints when compared to contact controls.

The inability of NfL to distinguish between SRC and pre-match levels in our study may be due to athletes being in-season and not pre-season as was used in the Shahim et al., 2018, study that did find differences between pre-season and post-SRC serum NfL levels at the one-hour timepoint [12]. NfL is thought to have a half-life of 3 weeks [28,32] and has shown to accumulate in players as an American football season progresses [32,33]. Therefore, the in-season rugby athletes in our study likely had elevated pre-game serum NfL levels, as they were playing at least one rugby match each week and may be a reason that there was not a statistically significant increase in serum NfL post-match or post-SRC compared to pre-match. For example, Shahim et al., 2018, showed preseason NfL levels an average of 10.8 pg·mL^−1^ vs. 15.7 pg·mL^−1^ in our study. The 15.7 pg·mL^−1^ baseline average in our study is more comparable to the 13.00 pg·mL^−1^ found in in-season American football players [32]. These data suggest that if NfL is to be used to aid in concussion diagnosis, baseline measures may need to be taken throughout a season. Although NfL was not found to distinguish between SRC and pre-match groups in our study, previous studies have found that NfL is better at distinguishing concussed from control individuals at much later time points (6–30 days) following concussion than the blood samples taken in our study, and may be more useful in concussion prognosis [34,35,36]. Therefore, NfL may be a better biomarker for aiding in the diagnosis of concussion at later time points rather than pitch-side.

An interesting finding from our study was that serum NfL was higher in the post-match group compared to the post-SRC group. It is possible, as we speculated with APP, that the greater playing time in the post-match athletes allowed more time after each subconcussive hit for serum NfL accumulation, whereas the early removal of SRC athletes from the match may not have permitted sufficient time for serum NfL accumulation. Indeed, as mentioned previously, serum NfL has been shown to accumulate as time from concussion increases.

VILIP-1 was significantly greater in the post-match group compared to the pre-match and SRC groups with no difference between the pre-match and SRC groups. This finding agrees with a previous study where a lack of significant increase 1 h post-SRC was found in concussed hockey players compared to pre-season baseline levels [11]. It was surprising, however, that the post-match group had greater serum VILIP-1 levels than both the pre-match and SRC groups. VILIP-1 has not been shown to increase significantly after high-intensity interval training (HIIT) exercise in cycling subjects [37], suggesting that subconcussive hits in the post-match group may have caused the increased VILIP-1 levels. On the other hand, previous research has also shown VILIP-1 to significantly increase as a result of playing a non-competitive game of hockey [11], suggesting that extracerebral sources of VILIP-1 such as the heart [38] and pancreas [39] may be responsible for the increases in VILIP-1 observed. However, it is unlikely that VILIP-1 from these extracerebral sources would increase in a friendly hockey game but not HIIT cycling exercise. Since it was not specified that the hockey athletes must refrain from contact in the non-competitive hockey game, it seems logical that subconcussive hits may have caused the increase in VILIP-1, supporting the hypothesis that VILIP-1 increase may be caused by subconcussive impacts. It is also possible that metabolic differences in the nature of exercise may cause changes in VILIP-1 as HIIT is largely non-aerobic and a non-competitive game of hockey may have a more aerobic exercise component as compared to HIIT or competitive hockey. Additional research into how VILIP-1 accumulates because of non-contact exercise and number of hits in a contact sport may help determine if exercise alone or subconcussive impacts cause an increase in serum VILIP-1 levels.

## 5. Conclusions

From a clinical standpoint, our study raises questions about whether APP, NfL, or VILIP-1 have diagnostic ability for assessing SRC. Although our sample size is small, these three proteins were unable to distinguish between concussed and non-concussed rugby players. APP, NfL, or VILIP-1 may have better clinically utility when post-SRC biomarker levels are compared to a pre-game or pre-season baseline; however, that was not investigated in this study.

There are various limitations in this study. The study enrolled a small sample size and did not include enough females to allow sex comparisons. A lack of paired data for the post-match athletes and SRC athletes adds a confounding variable since initial biomarker levels for the post-match and SRC athletes may have differed from the pre-match athletes included in the study. This may have played a role in our findings since the subjects making up each group, differed in sex, age, years of play, and concussion history. Collecting pre- and post-match blood samples from the same subjects may have also helped detect slight changes in biomarker levels due to subconcussion injury. The importance of using paired biomarker data to determine if biomarkers can be used to diagnose concussion can also be seen with the high interquartile ranges seen with our data, indicating high interindividual variability of biomarker concentration. Future studies should make a significant effort to collect paired data samples for better comparisons, along with including a much larger sample size as this will give better ability to detect smaller effect sizes which may be needed when investigating individual protein biomarkers. Including multiple timepoint blood draws as well as controlling for total play time and intensity of play for the post-match and SRC groups would have also added to the knowledge of how these biomarkers accumulate over time post-match and after SRC. We also did not record how long each athlete played in a match prior to being removed for concussion. This may have led to differences in biomarker accumulation as some concussed players may have played for a longer period of time than others and experienced a larger number of subconcussion injuries confounding biomarker accumulation. Another limitation is that we did not perform the SCAT5 on the post-match group and instead relied on the self-report of post-match athletes not experiencing concussion symptoms. There is a possibility that subjects in the post-match group could have been concussed, skewing the data. Future research should conduct SCAT5 assessment on the post-match group to ensure they do not meet clinical criteria for concussion. Another limitation in our study was the use of a standard ELISA to analyze NfL when the Simoa platform commonly used in other concussion studies would have provided greater sensitivity and may have allowed for the detection of subtle differences among groups. Using the Simoa platform would also have allowed our data to be more comparable to previous research investigating NfL.

Despite these limitations, our study adds to the current knowledge of how NfL and VILIP-1 accumulate in serum pitch-side and was the first to study APP in the serum of concussed athletes. We focused on obtaining blood samples pitch-side since concussion diagnosis is typically made on the sideline of athletic events. All three biomarkers were unable to differentiate concussed athletes from non-concussed athletes on their own in this small sample size. However, a troubling finding was that all three proteins increased significantly post-match, possibly suggesting that brain injury may occur due to subconcussive impacts experienced by rugby players. Therefore, it may be helpful if future research including these biomarkers use instrumentation to monitor quantity and magnitude of hits to determine if there is a relationship between biomarker accumulation and subconcussive hits. Including non-contact athlete controls pre- and post-exercise may also help distinguish if these biomarkers increase due to exercise in the absence of subconcussive hits.

## Figures and Tables

**Table 1:** Demographic data for all participants. Data reported as median (IQR) for self-reported history of concussion for each participant, as well as their years of playing rugby, age, and body mass. Participants were all Caucasian, and two participants were female. No significant difference was found among groups for concussion history (*p* = 0.082, η^2^ = 0.217), years of play (*p* = 0.143, η^2^ = 0.376), age (*p* = 0.119, η^2^ = 0.098), or body mass (*p* = 0.345, η^2^ = 0.043).

Group	Self-Reported Concussion History	Years of Play	Age (Years)	Body Mass (kg)
Pre-Match	0.00 (1.50)	2.00 (3.00)	21.00 (3.00)	85.91 (18.18)
Female (*n* = 0)				
Male (*n* = 9)				
Post-Match	2.00 (4.50)	3.00 (4.50)	22.00 (9.50)	90.45 (26.14)
Female (*n* = 2)				
Male (*n* = 19)				
SRC	3.00 (7.50)	1.50 (5.50)	23.50 (6.25)	82.95 (23.52)
Female (*n* = 0)				
Male (*n* = 7)				

**Table 2:** Area under the receiver operating characteristic curve (AUROC) comparing the different groups using amyloid beta precursor protein (APP). Values are represented as AUROC (95% confidence interval), *p*-value (*p*). SRC = Sports-related concussion. AUROC was converted to Cohen’s d using published conversion tables and formulas [17].

Between Group Comparisons	AUROC (95% CI), Cohen’s D, and *p*-Value for APP
SRC vs. Post-match	0.330 (0.115–0.545), d = −0.622, *p* = 0.185
SRC vs. Pre-match	0.556 (0.256–0.855), d = 0.199, *p* = 0.711
Post-match vs. Pre-match	0.709 (0.512–0.906), d = 0.778, *p* = 0.074

**Table 3:** Area under the receiver operating characteristic curve (AUROC) comparing the different groups using neurofilament light (NfL). Values are represented as AUROC (95% confidence interval), *p*-value (*p*). * = Significant difference between groups (*p* < 0.05). SRC = Sports-related concussion. AUROC was converted to Cohen’s d using published conversion tables and formulas [17].

Between Group Comparisons	AUROC (95% CI), Cohen’s d, and *p*-Value for NFL
SRC vs. Post-match	0.029 (0.000–0.094), d = −2.682 *p* < 0.001 *
SRC vs. Pre-match	0.127 (0.000–0.337), d = −1.613 *p* = 0.013 *
Post-match vs. Pre-match	0.852 (0.688–1.000), d = 1.478, *p* < 0.001 *

**Table 4:** Area under the receiver operating characteristic curve (AUROC) comparing the different groups using visinin-like protein-1 (VILIP-1). Values are represented as AUROC (95% confidence interval), *p*-value (*p*). * = Significant difference between groups (*p* < 0.05). SRC = Sports-related concussion. AUROC was converted to Cohen’s d using published conversion tables and formulas [17].

Between Group Comparisons	AUROC (95% CI), Cohen’s D, and *p*-Value for VILIP-1
SRC vs. Post-match	0.095 (0.000–0.246), d = −1.853, *p* = 0.003 *
SRC vs. Pre-match	0.444 (0.148–0.741), d = −0.199, *p* = 0.711
Post-match vs. Pre-match	0.970 (0.907–1.000), d = 2.661, *p* < 0.001 *

## Data Availability

The datasets used and analyzed during the current study are available from the corresponding author upon reasonable request.

## Supplementary Materials

The following supporting information can be downloaded at: https://www.mdpi.com/article/10.3390/sports10120194/s1, Table S1: APP comparison among groups; Table S2: APP paired data; Table S3: NfL comparison among groups; Table S4: VILIP-1 comparison among groups.
