# Supplementary material for: Serum Amyloid Beta Precursor Protein, Neurofilament Light, and Visinin-like Protein-1 in Rugby Players: An Exploratory Study
Source: Sports (Basel). 2022 Nov 29;10(12):194. doi: 10.3390/sports10120194 (PMC9782676; doi:10.3390/sports10120194)
Supplement: Supplementary file 1 [file sports-10-00194-s001.zip › Rogatzki_Tables_Suppl_R1.pdf]

Groups	APP (pg·mL^{-1})
Pre-match (n=9)	29.06 (75.60)
Post-match (n=21)	100.49 (118.50)
SRC (n=7)	38.20 (19.86)

Supplementary Table 1: Amyloid beta precursor protein (APP) concentration among groups. Subjects range from ages 18 to 60 years. All subjects were Caucasian, two subjects were female. Values are represented as median (IQR). SRC = sports-related concussion.

Group	Serum APP (pg·mL^{-1})
Pre-match (n=6)	57.98±63.21 (-8.36 – 124.31)
Post-match (n=6)	111.37±106.89 (-0.81 – 223.54)*

Supplementary Table 2: Descriptive data of amyloid beta precursor protein (APP) from the paired T-test statistical analysis. All subjects were Caucasian males. Values are represented as mean±SD (95% confidence interval). * = significantly different ($p<0.05$) compared to pre-match.

Groups	NfL ($pg \cdot mL^{-1}$)
Pre-match (n=9)	11.54 (9.60)
Post-match (n=15)	29.60 (57.45)
SRC (n=7)	8.71 (6.09)*

Supplementary Table 3: Serum neurofilament light (NfL) concentration among groups. Subjects range from ages 18 to 60 years. All subjects were Caucasian, two subjects were female. Values are represented as median (IQR) * = significantly different ($p<0.05$) compared to match-control. SRC = sports-related concussion.

Groups	VILIP-1 ($pg \cdot mL^{-1}$)
Pre-match (n=9)	32.63 (52.24)*
Post-match (n=15)	212.18 (345.00)
SRC (n=7)	30.21 (47.20)*

Supplementary Table 4: Serum visinin like protein-1 (VILIP-1) concentration among groups. Subjects range from ages 18 to 60 years. All subjects were Caucasian, two subjects were female. Values are represented as median (IQR). * = significantly different ($p<0.05$) compared to match-control. SRC = sports-related concussion.
